# Depression and anxiety consequences of the COVID-19 pandemic: A longitudinal cohort study with university students

**DOI:** 10.1192/j.eurpsy.2021.689

**Published:** 2021-08-13

**Authors:** V. Conceição, I. Rothes, R. Gusmão, H. Barros

**Affiliations:** 1 Epidemiology Research Unit (epiunit), Institute of Public Health of the University of Porto, Porto, Portugal; 2 Center For Psychology At University Of Porto, Portugal, Faculty of Psychology and Education Science, University of Porto, Porto, Portugal

**Keywords:** COVID-19, Depression, Anxiety, mental health

## Abstract

**Introduction:**

For young people, just as in the general population, COVID-19 caused many changes in their lives. The literature review has shown an increased risk for mental illness symptoms as a consequence of the pandemic.

**Objectives:**

With this study, we aimed to evaluate the impact of COVID-19 pandemic in university students’ anxiety and depression symptoms.

**Methods:**

This study is part of a larger longitudinal research on university students’ mental health with the Portuguese version of The Patient Health Questionnaire (PHQ-9) and the Portuguese version of the Generalised Anxiety Disorder (GAD-7) data with evaluations on January, May and October 2019 and June 2020, as well as socio-demographic information.

**Results:**

341 university students (257 females and 84 males) were included in this study, with a mean age of 19.91 (SD=1.58). In June 2020, the mean for perceived well-being loss was 60.47% (SD=26.56) and 59.54% (SD=28.95) for mental health loss. In the PHQ-9, the proportion of students with scores equal or above 15 ranged between 22.6% and 25.5% in 2019, however, in June 2020, the proportion was significantly higher (37.0%). The proportion of GAD-7 scores above the cut-off 10 ranged between 46.0% and 47.8% in 2019, and, in 2020, 64.5% of the students scored 10 or above. Compared with preceding trends, PHQ-9 scores were 3.11 (CI=2.40-3.83) higher than expected, and GAD-7 scores were 3.56 (CI=2.75-5.37) higher.

**Conclusions:**

COVID-19 had a negative impact on depressive and anxiety symptoms in university students, in line with the literature and confirming the vulnerability of young people in such uncertain times.

